# Expression of GPR68, an Acid-Sensing Orphan G Protein-Coupled Receptor, in Breast Cancer

**DOI:** 10.3389/fonc.2022.847543

**Published:** 2022-03-04

**Authors:** Noha Mousaad Elemam, Rana A. Youness, Amal Hussein, Israa Shihab, Nada M. Yakout, Yasmine Nagy Elwany, Tamer M. Manie, Iman M. Talaat, Azzam A. Maghazachi

**Affiliations:** ^1^Clinical Sciences Department, College of Medicine, University of Sharjah, Sharjah, United Arab Emirates; ^2^Sharjah Institute of Medical Research, University of Sharjah, Sharjah, United Arab Emirates; ^3^Biology and Biochemistry Department, School of Life and Medical Sciences, University of Hertfordshire Hosted by Global Academic Foundation, Cairo, Egypt; ^4^Department of Family and Community Medicine and Behavioral Sciences, College of Medicine, University of Sharjah, Sharjah, United Arab Emirates; ^5^Pathology Department, Faculty of Medicine, Alexandria University, Alexandria, Egypt; ^6^Clinical Oncology Department, Medical Research Institute, Alexandria University, Alexandria, Egypt; ^7^Department of Breast Surgery, National Cancer Institute, Cairo University, Cairo, Egypt

**Keywords:** GPR68, acidosis, tumor microenvironment, breast cancer, triple-negative breast cancer

## Abstract

Breast cancer (BC) is the most diagnosed cancer and the leading cause of global cancer incidence in 2020. It is quite known that highly invasive cancers have disrupted metabolism that leads to the creation of an acidic tumor microenvironment. Among the proton-sensing G protein-coupled receptors is GPR68. In this study, we aimed to explore the expression pattern of GPR68 in tissues from BC patients as well as different BC cell lines. Methods: In-silico tools were used to assess the expression of GPR68 in BC patients. The expression pattern was validated in fresh and paraffin-embedded sections of BC patients using qPCR and immunohistochemistry (IHC), respectively. Also, in-silico tools investigated GPR68 expression in different BC cell lines. Validation of GPR68 expression was performed using qPCR and immunofluorescence techniques in four different BC cell lines (MCF-7, MDA-MB-231, BT-549 and SkBr3). Results: GPR68 expression was found to be significantly increased in BC patients using the in-silico tools and validation using qPCR and IHC. Upon classification according to the molecular subtypes, the luminal subtype showed the highest GPR68 expression followed by triple-negative and Her2-enriched cells. However, upon validation in the recruited cohort, the triple-negative molecular subtype of BC patients showed the highest GPR68 expression. Also, in-silico and validation data revealed that the triple-negative breast cancer cell line MDA-MB-231 showed the highest expression of GPR68. Conclusion: Therefore, this study highlights the potential utilization of GPR68 as a possible diagnostic and/or prognostic marker in BC.

## Introduction

According to the World Health Organization (WHO), breast cancer (BC) is the most commonly diagnosed cancer and the leading cause of global cancer incidence in 2020, with an estimated 2.3 million new cases, representing 11.7% of all cancer cases. Furthermore, BC was reported to be the fifth leading cause of cancer-related mortality worldwide, with 685,000 deaths (1).

It is quite known that highly malignant and invasive cancers have disrupted metabolism and specifically an elevated glycolytic activity. This creates an acidic milieu, also known as the Warburg effect, which is an important hallmark of the tumor microenvironment (TME) (2). Such an environment regulates proliferation, apoptosis, and metastasis of cancer cells as well as modulate inflammation, anti-tumor immunity, and angiogenesis (3–5). Possible antagonizing approaches to this environment is the use of bicarbonate buffer that reduces growth and metastasis of cancers including melanoma, breast, prostate, pancreatic and lung cancers (6–8). Consequently, targeting tumor acidity may serve as a potential and promising therapeutic approach for cancers.

There are several acid-sensing cell surface receptors and ion channels that can sense acidity in the microenvironment; among them are proton sensing G protein-coupled receptors (GPCRs). GPCRs are considered the largest family of cell signaling receptors with over 800 GPCRs encoded in the human genome, representing approximately 3% of the human genome. They are seven-transmembrane spanning domain receptors that respond to numerous types of extracellular signals such as lipids, peptides, proteins, ions, and photons which regulate many physiological processes (9). Furthermore, GPCRs represent more than 30% of targets for FDA approved small molecules (10, 11). In tumors, GPCRs are known to regulate cellular processes that are critical for the initiation and progression of tumors, such as cell proliferation, inhibition of apoptosis, immune evasion, tumor invasion, angiogenesis, and metastasis (12, 13).

Among the members of the proton sensing GPCRs is GPR68, also known as ovarian cancer G protein-coupled receptor 1 (OGR1). It was first identified from the HEY human ovarian cancer cell line and is located on chromosome 14 band q31 (14q31) (14). So far, the only endogenous agonist of GPR68 is H^+^ ions/acidic environment, where it is inactive at pH 7.8 and becomes activated at pH 6.8 (15). Being coupled with Gαq subunit, GPR68 activation triggers Ca^2+^ release from intracellular stores, stimulates protein kinase C (PKC) signaling and formation of inositol trisphosphate (IP3). Moreover, GPR68 activates the mitogen-activated protein kinase (MAPK) signaling pathways (16–19). Also, GPR68 acts as a double‐edged sword, where it was found to be a tumor-suppressor in the prostate cancer (20), whereas other studies revealed that GPR68 has an oncogenic profile by promoting cancer outgrowth (21). In this study, we sought to investigate GPR68 expression in the breast tumor microenvironment that might aid in sensing acidosis and regulating BC progression.

## Subjects, Materials and Methods

### In-Silico Expression of GPR68 in Breast Cancer Patients and Cell Lines

In-silico tools TNM plot (https://www.tnmplot.com/) (22) and UALCAN TCGA data analysis (http://ualcan.path.uab.edu/index.html) (23) were used to assess the expression of GPR68/OGR1 in various cancers compared to normal tissues. Also, these tools were used to explore GPR68 expression in BC tissues compared to healthy ones. Moreover, the UALCAN tool was used to retrieve Kaplan-Meier plots in order to investigate if there is an association between GPR68 expression levels and the survival of BC patients. The UALCAN tool was also used to explore the association with the clinicopathological parameters of BC patients. On the other hand, GPR68 expression was explored in the different BC cell lines using the in-silico tool EMBL-EBI (https://www.ebi.ac.uk/gxa/home) by examining the data of RNA-seq in 934 human cancer cell lines from the cancer cell line encyclopedia.

### Breast Cancer Tissues

The cohort included in this study was composed of a total of 98 female Egyptian BC patients who underwent conservative breast surgery/mastectomy in Alexandria University, Kasr El-Aini and the National Cancer Institute hospitals, Egypt. Pathologists confirmed the pathological diagnosis of all samples, and their clinicopathological parameters were summarized in **Table 1**. The mean age (±SD) of recruited patients was 47.18 (±11.40) years. Some of the adjacent normal counterparts of the cancerous tissues were resected (n=15), that were used in the comparison with the fresh BC samples (n=28). Also, other non-tumor fibrocystic breast tissues (n=20) were collected for histological comparison to the formalin-fixed paraffin-embedded (FFPE) BC tissues (n=70). All patients enrolled in this study agreed and signed informed consents. The study was approved by the research ethics committee of the University of Sharjah, UAE (REC-21-09-04-01). All experiments were performed in compliance with the ethical standards of the declaration of Helsinki.

**Table 1:** Clinicopathological characteristics of the recruited cohort of breast cancer patients (n = 98).

Category	Frequency	Percent (%)
**Age**		
≤ 40	28	28.6
> 40	70	71.4
**Tumor size**		
T1	16	16.3
T2	50	51.0
T3	32	32.7
**Histologic type**		
Invasive ductal carcinoma	89	90.8
Invasive lobular carcinoma	5	5.1
Others	4	4.1
**Histologic grade**		
G1	8	8.2
G2	65	66.3
G3	24	24.5
G4	1	1.0
**ER status**		
Negative	47	48.0
Positive	51	52.0
**PR status**		
Negative	45	45.9
Positive	53	54.1
**Her2 status**		
Negative	78	79.6
Positive	20	20.4
**Ki-67**		
Low (*<*14)	26	26.5
High (≥14)	72	73.5
**Molecular subtype**		
Luminal A	33	33.7
Luminal B	24	24.5
Her2-enriched	9	9.2
Triple-negative	32	32.7
**Nodal status**		
N0	26	26.5
N1	32	32.7
N2	21	21.4
N3	19	19.4
**Tumor stage**		
Stage 1	9	9.2
Stage 2	36	36.7
Stage 3	53	54.1

### Cell Culture of Breast Cancer Cell Lines

Four different cell lines were used in the study, hormonal luminal A cell line (ER^+^, PR^+^, Her2^-^: MCF-7), triple-negative/basal-like cell lines (ER^-^, PR^-^, Her2^-^: BT-549 and MDA-MB-231), and the Her2^+^ SKBr3 (ER^-^, PR^-^, Her2^+^). All four cell lines were obtained from ATCC, USA. MCF-7, BT-549, MDA-MB-231 cell lines were cultured in complete RPMI-1640 medium, while SkBr3 was cultured in complete DMEM media. All culture media were supplemented with 2 mM L-glutamine, 1% non-essential amino acids, 100 U/mL penicillin, 100 μg/mL streptomycin, 71.5 μM 2-mercaptoethanol, and 10% fetal bovine serum (Sigma-Aldrich, St. Louis, MO, USA).

### RNA Extraction, Reverse Transcription, and qRT-PCR

Fresh breast tissues were snapped frozen in liquid nitrogen directly after collection and stored at -80°C. For RNA extraction from BC tissues, Trizol RNA extraction method was applied. For cell lines, RNA was extracted using the RNeasy extraction kit (Qiagen, Germany). Complementary DNA was synthesized using High-Capacity cDNA Reverse Transcription Kit (Thermofisher Scientific, USA). GPR68/OGR1 expression was detected and quantified using the primers (Forward: GTTTGAAGGCGGCAGAAATG, Reverse: GTGGAATGAGGAGGCATGAA), HOT FIREPol EvaGreen qPCR Supermix (SolisBioDyne, Estonia) and Quantstudio 3 real time qPCR (Applied Biosystems, USA). Ribosomal 18S was used as housekeeping gene and relative quantification was calculated as 2^-ΔΔCT^.

### Immunohistochemical Staining and Scoring of GPR68 Expression

BC paraffin-embedded tissues were sectioned at 4 μm, after which they were stained with rabbit anti-human GPR68 antibody (Invitrogen, USA, Cat. no: 720277), at 1:200 dilution. The secondary conjugation and detection were done using UltraVision Quanto Detection System HRP and DAB Quanto (Thermofisher Scientific, USA). The images were captured with Olympus DP74 microscope digital camera attached to a BX43 microscope (Olympus Life Sciences, Tokyo, Japan). Immunoreactive score (IRS) was used to evaluate the expression status of GPR68 in the different samples according to the recommendations by Remmele and Stegner (24). IRS is usually generated by the multiplication of the staining intensity and the percentage of immuno-stained cells with a range from 0-12. Microscopic evaluation of the immunohistochemical stainings was performed by two independent investigators. Also, semiquantitative analysis of DAB staining of GPR68 was done using the immunohistochemistry (IHC) Toolbox plugin in Image J software (https://imagej.nih.gov/ij/index.html). Optical density (OD) was calculated as log (max intensity/mean intensity).

### Immunofluorescence of GPR68 in Breast Cancer Cell Lines

The four different BC cell lines were seeded in 6 well plates, coated with cover slides. The cells were washed with PBS, fixed using 4% paraformaldehyde for 15 minutes and permeabilized using 0.1% Triton-X for 10 minutes. The cells were stained with the primary anti-human GPR68 antibody (Invitrogen, USA, Cat. no: 720277, 2 μg/ml), at 4°C and left overnight. Then, the secondary antibody AlexaFluor 488-conjugated goat anti-rabbit IgG (Invitrogen, USA, Cat. no: A-11008) was incubated for 45 minutes. After washing multiple times, coverslips were removed carefully and loaded on slides with DAPI nuclear stain (Invitrogen, USA). The images were captured with Olympus DP74 microscope digital camera attached to an BX43 inverted microscope (Olympus Life Sciences, Tokyo, Japan), at x400 and x1000. The blue color indicated the nucleus of the BC cell lines while the green color indicated the GPR68 expression.

### Statistical Analysis

Statistical analysis was performed using SPSS 27 (IBM, Armonk, NY, USA) software package and GraphPad Prism 6 (San Diego, CA, USA). For SPSS analysis, descriptive univariate analyses were conducted using frequencies and percentages for categorical variables as well as means, medians, and standard deviations for scale variables. The Chi-square test was performed to assess the associations between categorical variables. The normality of continuous variables was tested visually using the Q-Q plots and statistically using the Kolmogorov-Smirnov test. Differences in the means of normally distributed continuous variables were analyzed using the independent t-test and ANOVA test, for two independent or multiple samples, respectively. Non-parametric tests, including Mann–Whitney or Kruskal–Wallis tests were used for skewed continuous outcomes. For GraphPad Prism analyses, normality tests were conducted, and the non-parametric Mann Whitney U-test was used to compare two groups. P-value <0.05 was considered statistically significant.

## Results

### In-Silico Analysis of GPR68 Expression in Various Cancers

**Figure 1:**
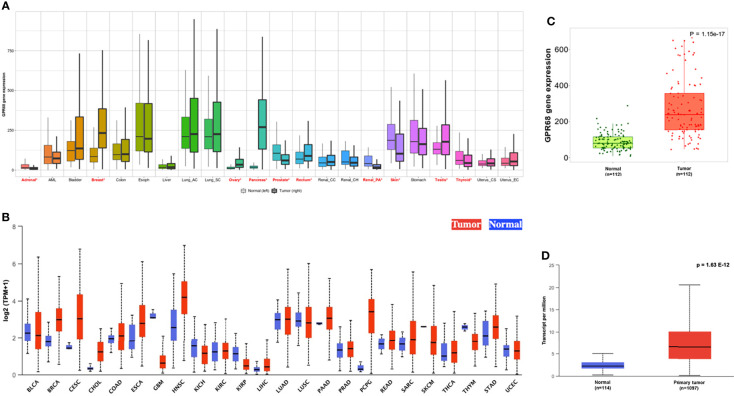
In-silico expression of GPR68 across various cancer types. **(A)** TNMplot showing breast cancer to be among the cancers where GPR68 was upregulated in tumor tissues. The significant differences by the Mann-Whitney U test are marked with “red*”. **(B)** UALCAN tool supporting GPR68 upregulation in breast cancer. **(C)** GPR68 was upregulated in the breast cancer tissues (n=112) compared to paired adjacent normal breast tissues using TNMplot data analysis. **(D)**UALCAN tool confirmed the upregulation pattern in 1097 breast cancer patients compared to 114 normal breast tissues. BLCA, Bladder urothelial carcinoma; BRCA, Breast invasive carcinoma; CESC, Cervical squamous cell carcinoma and endocervical adenocarcinoma; CHOL, Cholangiocarcinoma; COAD, Colon adenocarcinoma; ESCA, Esophageal carcinoma; GBM, Glioblastoma multiforme; HNSC, Head and neck squamous cell carcinoma; KICH, Kidney chromophobe; KIRC, Kidney renal clear cell carcinoma; LIHC, Liver hepatocellular carcinoma; LUAD, Lung adenocarcinoma; LUSC, Lung squamous cell carcinoma; PAAD, Pancreatic adenocarcinoma; PCPG, Pheochromocytoma and paraganglioma; PRAD, Prostate adenocarcinoma; READ, Rectum adenocarcinoma; SARC, Sarcoma; SKCM, Skin cutaneous melanoma; STAD, Stomach adenocarcinoma; THCA, Thyroid carcinoma; THYM, Thymoma; UCEC , Uterine corpus endometrial carcinoma.

GPR68 expression was assessed across various cancer types using the online tools TNMplot (https://www.tnmplot.com) and UALCAN (http://ualcan.path.uab.edu/index.html). As shown in **Figure 1A**, the TNMplot tool explored the GPR68 expression in various cancer types, where BC was among the tumors with a significant differential expression. This expression pattern was also reported in the UALCAN tool data (**Figure 1B**). In particular, as shown in **Figure 1C**, BC tumor tissue showed higher GPR68 expression compared to adjacent normal tissues (p=1.15e-17). This was further validated by UALCAN tool where a similar expression pattern was observed in BC patients (n=1097) that showed significantly higher GPR68 compared to normal breast tissues (n=114) with a p-value of 1.63e-12 (**Figure 1D**).

### Breast Cancer Patients’ Survival Based on GPR68 Expression

**Figure 2:**
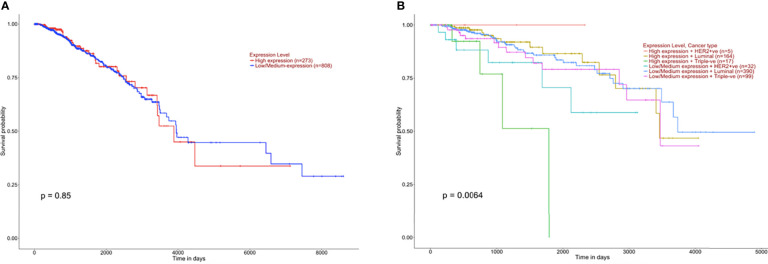
Kaplan Meier survival graphs of breast cancer patients based on GPR68 expression using UALCAN in-silico tool. **(A)** Survival plot of breast cancer patients that are classified according to high and low GPR68 expression showing that it is not a prognostic factor. **(B)** Classification of breast cancer patients with low and high GPR68 expression as well as molecular subtypes (Her2-enriched, luminal and triple-negative) showed GPR68 to be a potential marker affecting the survival of breast cancer patients.

It was crucial to explore whether GPR68 might have any effect on the prognosis and survival of BC patients. To investigate this issue, in-silico UALCAN tool was implemented. The results showed that GPR68 was not a potential prognostic factor in BC (p=0.85, **Figure 2A**). However, upon the classification of patients according to the molecular subtypes, luminal, Her2-enriched or triple-negative, GPR68 showed a significant effect on BC patients’ survival (p=0.0064, **Figure 2B**).

### Validation of GPR68 mRNA and Protein Expression in Breast Cancer Patients

**Figure 3:**
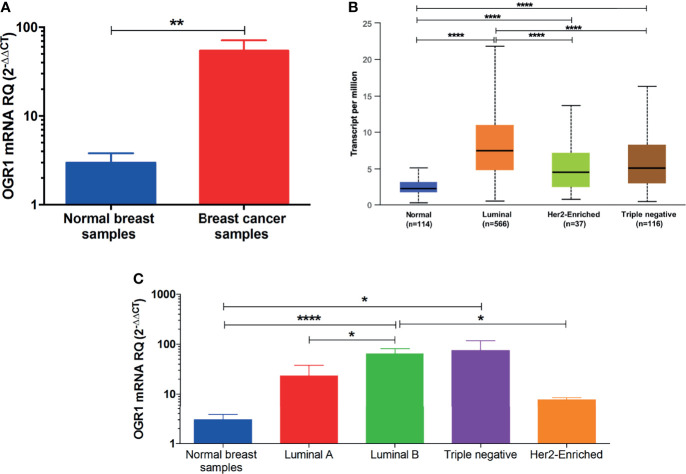
Quantification of GPR68 mRNA expression in breast cancer patients using qRT-PCR. **(A)** GPR68 mRNA expression was upregulated in breast cancer tissue samples of the recruited cohort compared to their normal counterparts. **(B)** In-silico analysis of GPR68 among the various molecular subtypes of breast cancer. **(C)** Validation of GPR68 expression on the mRNA level in the luminal A, luminal B, Her2-enriched and triple-negative breast cancer subtypes. *p<0.05, **p<0.01, ****p<0.0001.

The in-silico data was validated in BC patients’ samples collected from different hospitals. As illustrated in **Figure 3A**, the mRNA of GPR68 expression was higher in BC patients compared to normal breast tissues (p<0.01). Further, the in-silico data revealed that GPR68 is differentially expressed in the various molecular subtypes of BC. High GPR68 expression was found to be in the luminal as well as the triple-negative molecular subtype, as shown in **Figure 3B**. To confirm these observations, quantitative real-time qPCR performed on our cohort showed that triple-negative and luminal B subtypes had high expression of GPR68 expression as compared to normal controls (**Figure 3C**, p<0.05 and p<0.0001, respectively).

**Figure 4:**
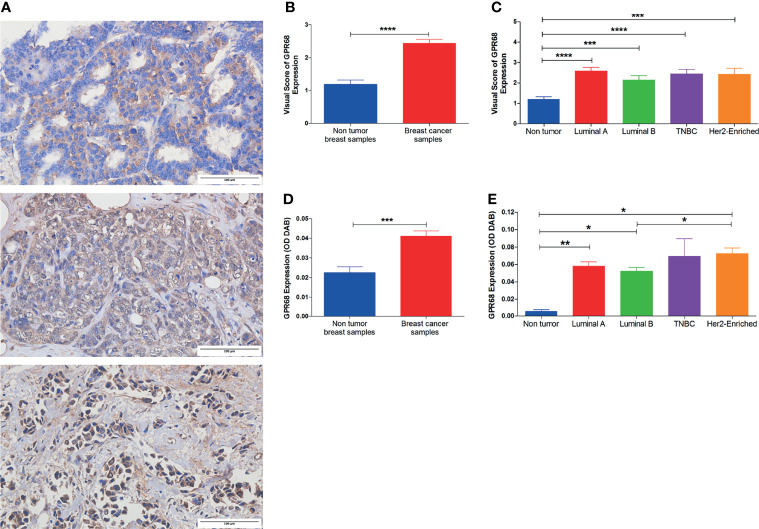
Immunohistochemical assessment of GPR68 expression in paraffin-embedded breast cancer tissues. **(A)** Microscopic images showing various degrees of intensity: mild, moderate, and strong GPR68 expression. Images were captured at x400 magnification, with a scale bar representing 100 μm. Brown/DAB staining denotes GPR68 expression. **(B)** Immunoreactive scoring of GPR68 expression in immunohistochemical staining of BC tissues compared to non-tumor tissues. **(C)** GPR68 expression according to immunoreactive scores between the different molecular subtypes of breast cancer patients. **(D)** Semi-quantitative assessment of GPR68 expression in breast cancer patients compared to non-tumor breast tissues, by calculating the optical density of DAB substrate using IHC toolbox-Image J. **(E)** Semi-quantitative assessment of GPR68 expression across the different molecular subtypes of breast cancer patients. *p<0.05, **p<0.01, ***p<0.001, ****p<0.0001.

In addition, GPR68 expression was validated in the recruited cohort and assessed by immunohistochemical staining of paraffin-embedded BC tissues. Different intensities were observed in BC tissues ranging from mild, moderate, to strong staining, with a cytoplasmic and/or membranous localization (**Figure 4A**). All BC sections were scored using the IRS system, that is usually generated by the multiplication of the staining intensity and the percentage of immuno-stained cells with a range from 0-12 (24).GPR68 was found to be higher in BC samples when compared to non-tumor breast tissues (**Figure 4B**). As shown in **Figure 4C**, upon the classification of BC patients, GPR68 expression in BC tissues showed a high expression in all the molecular subtypes. Such an expression pattern was further confirmed using the semi-quantification method via IHC Toolbox by Image J, where a higher expression of GPR68 was observed in BC tissues compared to non-tumor breast samples (**Figure 4D**). The expression across the different molecular subtypes was compared where the highest expression was observed in the triple-negative BC group, followed by the Her2-enriched and subsequently the luminal subgroup (**Figure 4E**). A negative control image as well as representative images of GPR68 expression in the different molecular subtypes of BC are illustrated in **Figure S1**.

### Association of GPR68 Expression With the Clinicopathological Parameters of Breast Cancer Patients

It was important to assess the correlation between GPR68 expression and the clinicopathological parameters of BC patients, in the in-silico data and recruited cohort (**Table 2**). The in-silico data revealed GPR68 expression to be unaltered across the different age groups. This was similar to the association reported from our recruited cohort, where age was not found to affect GPR68 expression in BC patients. Upon investigating the association with the molecular subtypes of BC, the in-silico data showed that the luminal group had a higher expression as compared to the Her2-enriched and triple-negative patients (p<0.0001 for both). However, in our recruited cohort the semi-quantification of GPR68 expression was higher in non-hormonal BC patients (triple-negative and Her2-enriched) when compared to the hormonal luminal A and B (p=0.046). This was further supported by a higher GPR68 expression in the PR negative BC patients as compared to PR positive BC patients (p<0.05). Lastly, in-silico data reported a significant change according to nodal metastasis status, where BC patients with N1 and N2 profiles had higher GPR68 expression compared to those with N0 (p<0.05). However, this was not observed in the recruited cohort.

**Table 2:** Association between GPR68 expression and the clinicopathological parameters of breast cancer patients, using data from the in-silico and the recruited cohort in the study.

	In-silico		Recruited Cohort		
	Categories	p value	Categories	GPR68 expression	p value
**Age**	21-40 yrs vs. 41-60 yrs	0.887	≤ 40	0.033492	0.782
	21-40 yrs vs. 61-80 yrs	0.832			
	21-40 yrs vs. 81-100 yrs	0.999			
	41-60 yrs vs. 61-80 yrs	0.897	> 40	0.044524	
	41-60 yrs vs. 81-100 yrs	0.91			
	61-80 yrs vs. 81-100 yrs	0.866			
**Tumor Stage**	Stage 1 vs. Stage 2	0.851	Early (1–2)	0.044313	0.308
	Stage 1 vs. Stage 3	0.24			
	Stage 1 vs. Stage 4	0.376			
	Stage 2 vs. Stage 3	0.105	Advanced (3–4)	0.040389	
	Stage 2 vs. Stage 4	0.381			
	Stage 3 vs. Stage 4	0.766			
**Molecular Subtype**	Luminal vs. Her2-enriched	0.000002****	Non-hormonal: Triple-negative & Her2-enriched	0.048693	0.046*
	Luminal vs. triple-negative	0.000078****			
			Hormonal: Luminal A & B	0.027408	
	Her2-enriched vs. triple-negative	0.209			
**Nodal Metastasis Status**	N0 vs. N1	0.011*	Negative	0.042336	0.529
	N0 vs. N2	0.045*			
	N0 vs. N3	0.813			
	N1 vs. N2	0.764	Positive	0.038511	
	N1 vs. N3	0.241			
	N2 vs. N3	0.233			

Bold text indicates significant findings. *p<0.05 and ****p<0.0001.

**Figure 5:**
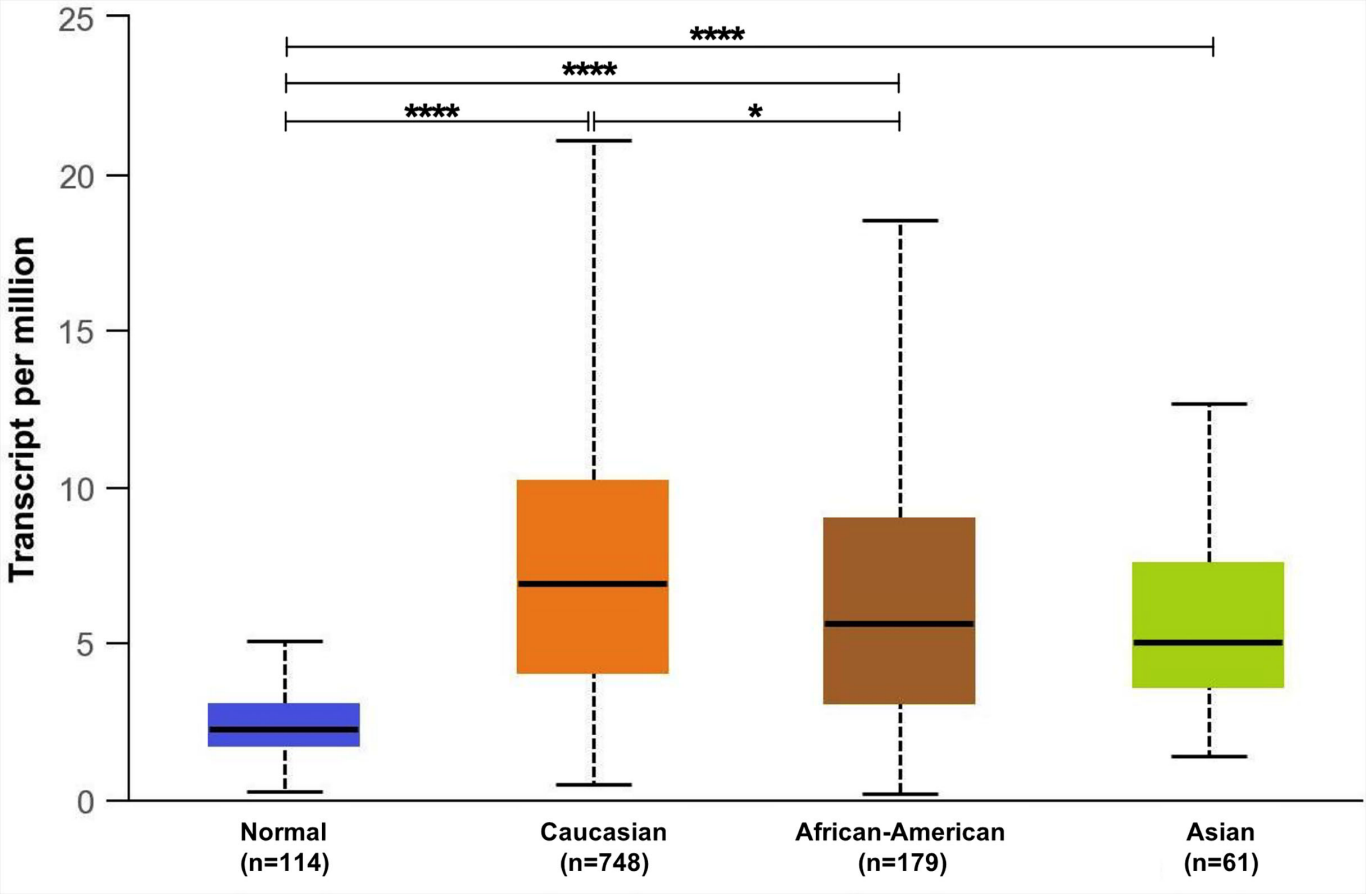
In-silico analysis of GPR68 expression across the different ethnicities (Caucasians, African-American and Asians) of breast cancer patients compared to normal breast samples. *p<0.05 and ****p<0.0001.

The observed discrepancy between the in-silico and validated GPR68 expression could be attributed to the different ethnicities between the BC patients. As mentioned earlier, our recruited cohort is comprised of Egyptian BC patients, i.e., African ethnicity, while the in-silico data was mainly composed of Caucasians, African Americans, and Asians. Intriguingly, the in-silico UALCAN tool showed that there was a significant increase in GPR68 expression in the Caucasian population in comparison to the African American population (p<0.05, **Figure 5**). This highlights the impact of race and ethnicity on GPR68 expression in BC.

### Validation of GPR68 mRNA and Protein Expression in Breast Cancer Cell Lines

**Figure 6:**
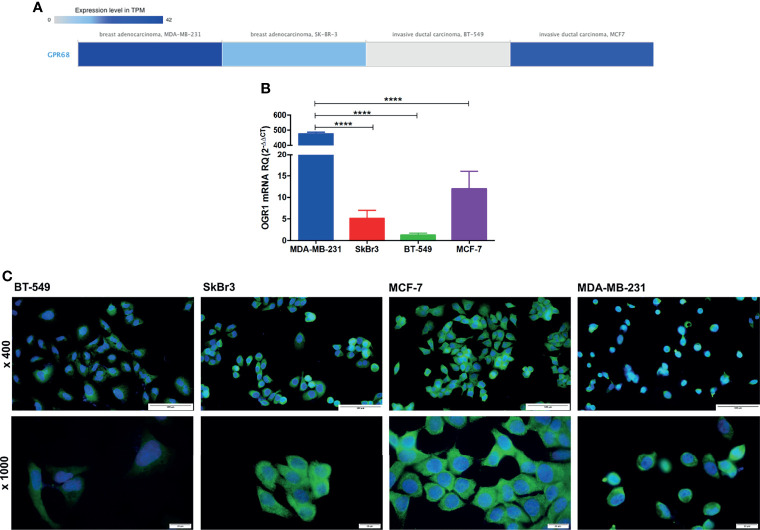
GPR68 expression in breast cancer cell lines. **(A)** In-silico data showing GPR68 expression in 4 different breast cancer cell lines using the EMBL-EBI tool. The grey color represents the lowest expression while the darkest blue color represents the highest expression. **(B)** Validation of GPR68 mRNA expression using qPCR in breast cancer cell lines with MDA-MB-231 showing the highest expression. **(C)** Immunofluorescence of GPR68 in breast cancer cell lines, where the blue color indicates the nucleus, and the green fluorescence represents GPR68 expression. Microscopic images were captured at x400 and x1000 magnification, with a scale bar of 100 and 20 μm, respectively. ****p<0.0001.

In order to assess the effect and mechanism of GPR68 in BC, four cell lines were selected as they showed various GPR68 expression according to the EBI tool using RNA-seq data of cancer cell line encyclopedia (**Figure 6A**). Upon validation of this data with qPCR, the triple-negative adenocarcinoma MDA-MB-231 showed the highest expression of GPR68 at the mRNA level, followed by the luminal A MCF-7 cell line, followed by Her2^+^ SkBr3 and lastly the triple-negative invasive ductal carcinoma BT-549 (**Figure 6B**). This was further validated using immunofluorescence, where a cytoplasmic and membranous expression of GPR68 was observed (**Figure 6C**).

## Discussion

In this study, GPR68 expression in BC was explored using various approaches including in-silico analysis, fresh biopsies, FFPE tissues, and cell lines. A significant upregulation pattern was observed along with a differential expression in BC molecular subtypes, suggesting a potential role in BC pathogenesis that needs to be further studied.

The effects of acidosis on cancer cells have been previously investigated in different tumors (3). However, the exact mechanisms and receptors that might facilitate these effects still need to be further explored. Among the proton sensing GPCRs is OGR1/GPR68, which is considered a novel pH sensor that is activated by an acidic extracellular pH (15, 19). Such proton sensing GPCRs were reported to play a role in tumor development, metastasis, inflammation, and angiogenesis process (3). GPR68 expression has been investigated across cancer types including skin, head and neck squamous cancer as well as pancreatic ductal adenocarcinoma (25–28). Since BC is the most prevalent cancer globally, we aimed at investigating the expression pattern of GPR68 in order to understand its role in the BC microenvironment.

Our in-silico data revealed GPR68 expression to be highly upregulated in BC across the different tumor types. This goes in line with previous in-silico findings by Wiley et al. where the most prominent increased GPR68 expression was in pancreatic ductal adenocarcinoma, cervical squamous cell carcinoma, certain subtypes of breast adenocarcinoma and ovarian cancer (29). The in-silico data revealed a high transcript level of GPR68 BC patients as compared to normal breast samples. This was further confirmed at the mRNA and protein levels by qPCR and IHC, respectively in the recruited cohort. Furthermore, GPR68 didn’t show any prognostic potential in BC patients unless they were classified according to their molecular subtypes, which is similar to the findings reported by Zhang et al. where the high GPR68 expression group did not have different survival rates (25). The in-silico data revealed that luminal subtypes have the highest GPR68 expression, followed by the triple-negative and Her2-enriched BC subtypes. Nevertheless, upon validation of GPR68 expression at the mRNA and protein levels, it was observed that the highest expression is in the triple-negative molecular subtype. Furthermore, there was a higher GPR68 expression in the PR negative BC patients when compared to the PR positive BC patients. Such discrepancy between the in-silico and validated GPR68 expression could be attributed to the different ethnicities between the BC patients, which was further supported by GPR68 expression across different ethnicities using the in-silico UALCAN tool. Such findings point out the effect of race and ethnicity on GPR68 expression, especially in BC.

Previous studies demonstrated the role of GPR68 in tumor development where GPR68 deficiency significantly reduced tumor allograft development in GPR68 knockout mouse model of prostate cancer cells (21). In addition, activation of GPR68 caused the stimulation and secretion of proinflammatory mediators such as IL‐6 and IL‐8 (CXCL8), which triggered tumor progression (30–32). The expression of GPR68 in BC cell lines was previously reported by Herzig et al., which showed a weak GPR68 expression in the BC cell lines MCF-7 and MDA-MB-231 (27, 33). Since the molecular subtype was found to affect GPR68 expression, it was essential to explore the baseline expression of GPR68 in four different BC cell lines. In-silico data, as well as the mRNA and protein expression of GPR68, revealed a strong expression in the triple-negative adenocarcinoma MDA-MB-231 cell line, followed by the luminal A MCF-7 cell line, and Her2^+^ SKBr3, with the lowest expression existing in the invasive ductal carcinoma triple-negative BT-549 cell line. Additionally, our data indicated a membranous and cytoplasmic GPR68 expression that could be possibly due to the internalization of GPR68 that might occur as a consequence of excessive activation, as previously reported (34). Such a process would need additional confirmation in future functional studies. Previous studies utilized MCF-7 cell lines to investigate the role of GPR68 in BC, where its overexpression inhibited cell migration by a Gα12/13-Rho-Ras-related C3 botulinum toxin substrate 1 (Rac1) pathway (33). Furthermore, overexpression of GPR68 increased the apoptosis of MCF-7 BC cells and inhibited cell growth, migration, and proliferation (33, 35). Our data revealed that MDA-MB-231 is a good candidate to investigate the function of GPR68 in BC.

In conclusion, this study is the first to report GPR68 expression in BC patients and its association with the clinicopathological parameters including molecular subtypes. Moreover, this study explores GPR68 expression across various cell lines showing MDA-MB-231 as a potential candidate for further studies to explore GPR68 in the BC microenvironment and allow researchers to understand its role in the pathogenesis of BC.

## Data Availability Statement

The original contributions presented in the study are included in the article/**Supplementary Material**. Further inquiries can be directed to the corresponding authors.

## Ethics Statement

The studies involving human participants were reviewed and approved by Research ethics committee of the University of Sharjah, UAE (REC-21-09-04-01). The patients/participants provided their written informed consent to participate in this study.

## Author Contributions

Conceptualization, NE, IT, and AM. Methodology, NE and IS. Formal analysis, NE and AH. Investigation, NE and IT. Data curation, RY, NY, YE, and TM. Writing—original draft preparation, NE. Writing—review and editing, RY, AH, IT, and AM. Supervision, IT and AM. All authors have read and agreed to the final version of the manuscript.

## Funding

This research was funded by the University of Sharjah, Sharjah, UAE, grant number 1901090255.

## Conflict of Interest

The authors declare that the research was conducted in the absence of any commercial or financial relationships that could be construed as a potential conflict of interest.

## Publisher’s Note

All claims expressed in this article are solely those of the authors and do not necessarily represent those of their affiliated organizations, or those of the publisher, the editors and the reviewers. Any product that may be evaluated in this article, or claim that may be made by its manufacturer, is not guaranteed or endorsed by the publisher.

## Supplementary Material

Supplementary Figure 1 Representative images of immunohistochemical staining of breast cancer tissues. **(A)** A negative control for the immunohistochemical staining process. **(B)** Representative images of GPR68 expression in luminal A, luminal B, Her2-enriched and triple-negative breast cancer tissues. All images were captured at x200 magnification, with a scale bar of 200 μm.
