# Supplementary figures and images for: Expression of GPR68, an Acid-Sensing Orphan G Protein-Coupled Receptor, in Breast Cancer
Source: Front Oncol. 2022 Mar 4;12:847543. doi: 10.3389/fonc.2022.847543 (PMC8930915; doi:10.3389/fonc.2022.847543)

**A.**

**Negative Control**

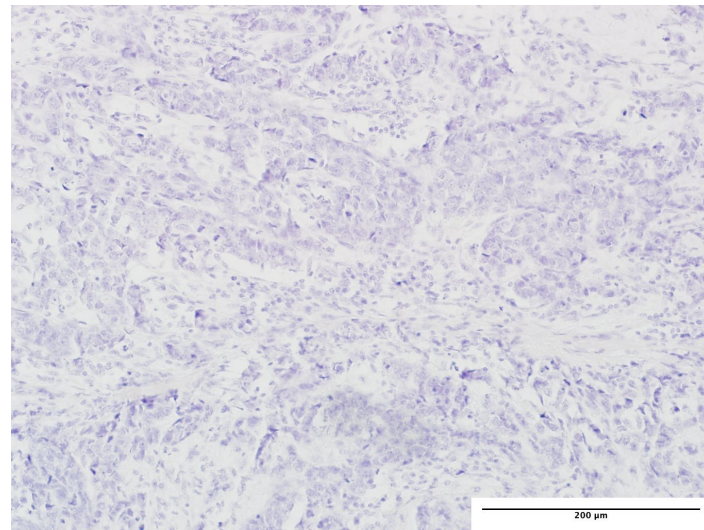

**B.**

**Luminal A**

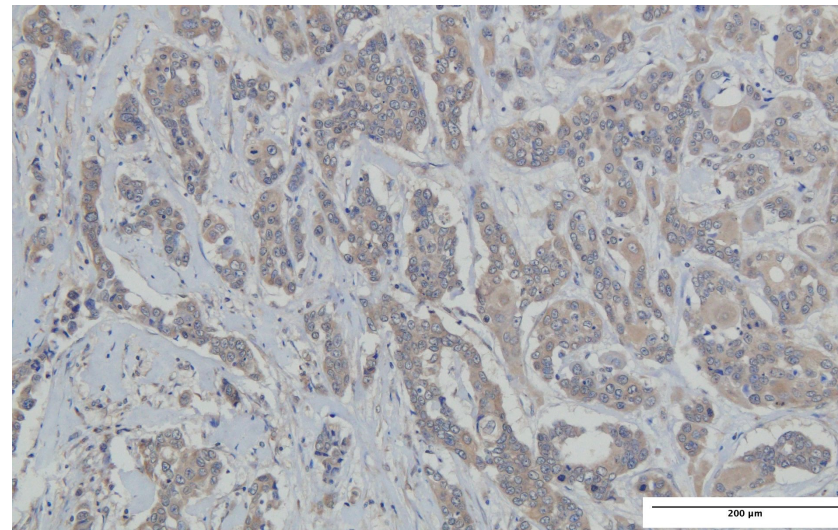

**Luminal B**

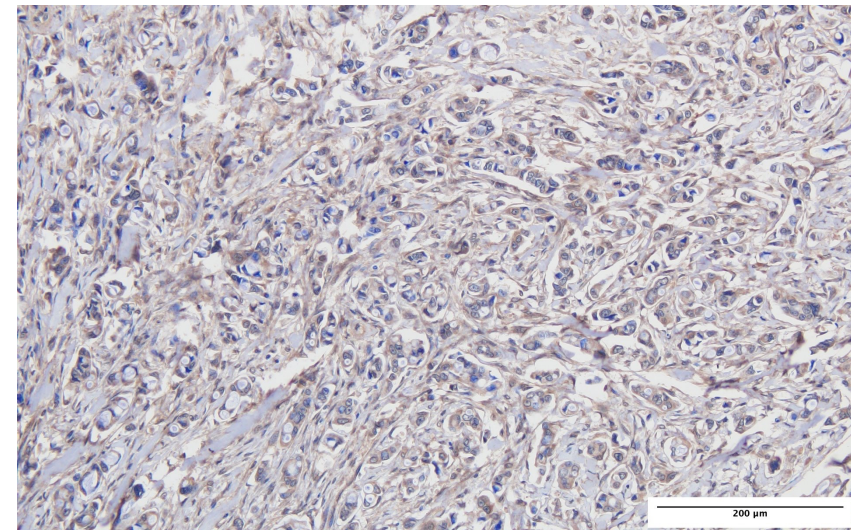

**Her2-enriched**

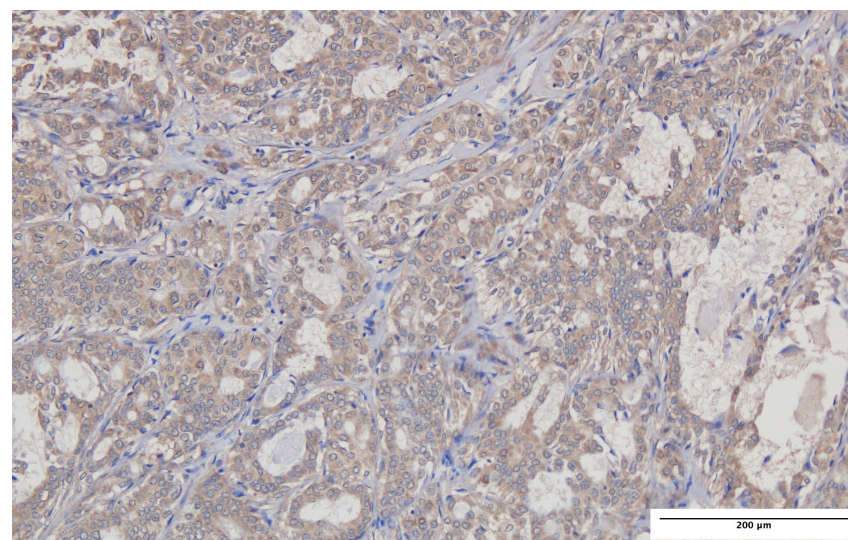

**Triple-negative**

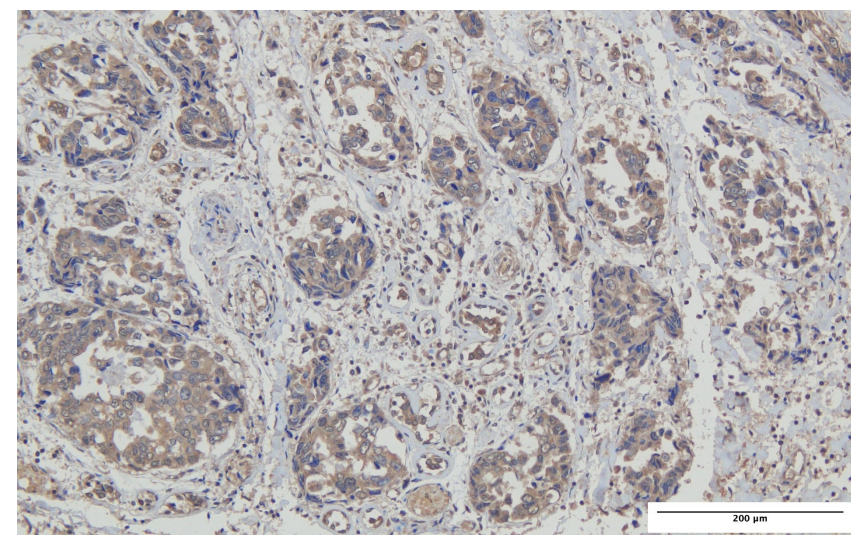

Supplement: Supplementary Figure 1 — Representative images of immunohistochemical staining of breast cancer tissues. (A) A negative control for the immunohistochemical staining process. (B) Representative images of GPR68 expression in luminal A, luminal B, Her2-enriched and triple-negative breast cancer tissues. All images were captured at x200 magnification, with a scale bar of 200 μm. [file Image_1.pdf]
